# Development of an Ultra-Dense Genetic Map of the Sunflower Genome Based on Single-Feature Polymorphisms

**DOI:** 10.1371/journal.pone.0051360

**Published:** 2012-12-19

**Authors:** John E. Bowers, Savithri Nambeesan, Jonathan Corbi, Michael S. Barker, Loren H. Rieseberg, Steven J. Knapp, John M. Burke

**Affiliations:** 1 Department of Plant Biology, University of Georgia, Athens, Georgia, United States of America; 2 Department of Ecology and Evolutionary Biology, University of Arizona, Tucson, Arizona, United States of America; 3 Department of Botany, University of British Columbia, Vancouver, British Columbia, Canada; 4 Department of Biology, Indiana University, Bloomington, Indiana, United States of America; 5 Center for Applied Genetic Technologies, University of Georgia, Athens, Georgia, United States of America; University of New England, Australia

## Abstract

The development of ultra-dense genetic maps has the potential to facilitate detailed comparative genomic analyses and whole genome sequence assemblies. Here we describe the use of a custom Affymetrix GeneChip containing nearly 2.4 million features (25 bp sequences) targeting 86,023 unigenes from sunflower (*Helianthus annuus* L.) and related species to test for single-feature polymorphisms (SFPs) in a recombinant inbred line (RIL) mapping population derived from a cross between confectionery and oilseed sunflower lines (RHA280×RHA801). We then employed an existing genetic map derived from this same population to rigorously filter out low quality data and place 67,486 features corresponding to 22,481 unigenes on the sunflower genetic map. The resulting map contains a substantial fraction of all sunflower genes and will thus facilitate a number of downstream applications, including genome assembly and the identification of candidate genes underlying QTL or traits of interest.

## Introduction

Cultivated sunflower (*Helianthus annuus*) is one of the world's most important oilseed crops, and also serves as an important source of confectionery seeds and cut flowers. Though numerous genetic maps of the sunflower genome have been produced (e.g., [1]–[4]; http://www.sunflower.uga.edu/cmap), the majority of these contain relatively few mapped loci. While maps with 250–300 loci have proven sufficient for defining the haploid set of 17 chromosomes and for localizing quantitative trait loci (QTLs), detailed comparative genomic analyses and whole genome sequence assemblies would benefit from the availability of much denser maps. More recently, a consensus genetic map containing ca. 10,000 loci was produced [5]. Given the extremely large size of the sunflower genome (ca. 3.6 billion bp), however, even higher marker densities are desirable.

The sunflower consensus map [5] was based on data from four different mapping populations that were genotyped with a high-density Illumina single nucleotide polymorphism (SNP) array [6]. While such tools produce high quality data, the requirement that SNPs be pre-identified through the re-sequencing of specific genotypes limits the utility of this approach if the goal is to produce an extremely high-density map from a particular population. Given the ability to produce oligonucleotide arrays containing millions of features, microarray-based genotyping provides a cost-effective means for simultaneously testing huge numbers of short probes for single-feature polymorphisms (SFPs; i.e., SNPs or indels that influence hybridization intensity; [7]) that can be used to construct an ultra-dense genetic map based on sequence-tagged markers. The consequence of such approaches are new computational challenges related to both the quantity and quality of the resulting data.

Genetic map construction is an NP-hard problem similar to the traveling salesman problem [8], in which a relatively simple ordering problem becomes increasingly complex with additional nodes (or loci), rapidly exceeding the complexity that even very powerful computers can solve through an exhaustive search. With the advent of microarray-based genotyping methods, as well as those based on the extraction of genotypic information directly from next-generation sequence data, it is becoming possible and economically practical to genotype tens of thousands to even millions of polymorphisms within a single genetic mapping population. Such large datasets require re-thinking the computational approach used to construct genetic maps. However, given the availability of a well-characterized, advanced generation mapping population, this problem can be resolved through an approach analogous to bin mapping [9], albeit at much higher resolution.

One of the best characterized sunflower mapping populations is a set of recombinant inbred lines (RILs) derived from a cross between sunflower cultivars RHA280 and RHA801. This population, which served as the basis of several early mapping efforts [2], [3], [10], was used to establish a universal system of sunflower chromosomal nomenclature that is now widely employed across research groups. The RHA280×RHA 801 RILs also played a crucial role in the development of the consensus map described above [5]. As part of that work, a total of 3785 SNPs were mapped in this population, resulting in a map spanning the expected 17 linkage groups with an average of nearly 3 markers per centimorgan (cM). Due to the high level of genomic coverage and extremely low error rates in that dataset, new markers can be added to this map simply by matching their segregation patterns to those observed for previously mapped loci or by finding an interval between two such loci into which the new marker can be placed without requiring additional recombination events.

Herein, we describe the genotypic characterization of a subset of the RHA280×RHA801 mapping population using a custom Affymetrix GeneChip. We used these data to map tens of thousands of SFPs corresponding to a substantial fraction of all genes in the sunflower genome.

## Materials and Methods

### Design of the Affymetrix Array

All genotyping was performed using a custom Affymetrix GeneChip (Affymetrix, CA, USA) designed from *Helianthus* expressed-sequence tags (ESTs). A total of 283,605 Sanger ESTs from 7 sunflower species (GenBank numbers: AJ318230–AJ318330, AJ412174–AJ412667, AJ437699–AJ437975, AJ539583–AJ540226, AJ541055–AJ541795, AJ542101–AJ542392, AJ827751–AJ829440, BG734514–BG734530, BG874297–BG874313, BG891021–BG891022, BQ909263–BQ917261, BQ965129–BQ980049, BU015365–BU036497, BU671782–BU672110, CD845604–CD858495, CF076145–CF099271, CX943504–CX948070, DY903733–DY959228, EE605695–EE627562, EE628472–EE661299, EL412382–EL492411, EL511146–EL515442, EL772988) were assembled using TGICL [11]. These sequences included 94,017 ESTs from *H. annuus*; 35,704 from *H. argophyllus*; 21,589 from *H. ciliaris*; 33,959 from *H. exilis*; 30,504 from *H. paradoxus*; 27,479 from *H. petiolaris*; and 40,353 from *H. tuberosus*. The final assembly included 87,237 “unigenes” corresponding to 27,587 contigs and 59,650 singletons (data deposited in the Dryad repository: http://dx.doi.org/10.5061/dryad.h0jg4).

Between 1 and 196 oligos corresponding to 25 bp sequences from each of 86,023 unigenes were included in the Affymetrix chip design; the remaining unigenes were either very short, consisted largely of repetitive simple sequences, or were redundant with other sequences already on the chip. This resulted in a chip design containing a total of 2,372,825 features from the *Helianthus* genome. The vast majority of the unigenes (84,916) were represented by 7 or more non-overlapping 25 bp probes. An additional 16,773 random 25 bp features were included on the chip as controls, along with 317 features from 8 different *Helianthus annuus* genomic (i.e., non-EST) sequences. The final chip design contained a total of 2,598,544 unique 25 bp features including 218,630 positive and negative controls (i.e., blank features and features matching a control non-sunflower DNA that was added to the labeling mix).

### Plant Materials

Genetic mapping was performed using 69 lines from an 8^th^ generation RIL population derived from a cross between sunflower RHA280×RHA801 (see [10] for details). RHA280 is a public confectionery restorer line whereas RHA801 is a public oilseed restorer line [12], [13]. As noted above, this population has been the subject of extensive investigations [2], [5], [10], [14].

### DNA Isolation, Labeling, and Microarray Hybridization

Total genomic DNA was extracted from a single plant per line using a CTAB extraction protocol [15]. 30 µg of DNA was digested using RQ1 RNase-Free DNase (Promega, WI, USA) to obtain fragments ranging in size from 50–300 bp. The fragmented DNA was labeled using GeneChip DNA Labeling Reagent. This was followed by hybridization of the labeled target to the sunflower array using the protocol outlined in the Affymetrix GeneChip Whole Transcript (WT) Double-Stranded Target Assay Manual. Hybridization was performed for 16–18 hours at 45°C and 60 rpm using an Affymetrix GeneChip Hybridization Oven 640. The washing and staining of the array was performed using the protocol provided by GeneChip WT Double-Stranded Target Assay Manual (FS450_0001/FS450_0002) using a GeneChip Fluidics Station 450. Chips were then scanned using an Affymetrix GeneChip Scanner 3000 7G. Each of the 69 RILs included in this study were tested in replicate on separate chips and the parents of the cross were each tested 4 times.

### Data Processing

The raw data for all chips were normalized via quantile normalization using Affymetrix Power Tools (APT v1.14.2). The normalized chip files were then converted to text using the APT software and the average intensity across all chips for each feature was computed using a custom script. For each chip, any feature with an intensity level <92% of the average for that feature on all chips was tentatively declared to represent genotype “AA”, while any intensity level >98% of the average was declared to represent genotype “BB”. Features on each chip with intensity levels 92–98% of average for that feature on all chips were declared as unknown. The 92% and 98% thresholds were determined empirically by studying a subset of features that appeared to show approximately 1∶1 segregation and good reproducibility between replicates. The initial calls for the two replicates of the same RIL were combined to create a consensus. If the two replicates had different genotype calls or were both “unknown”, then the consensus call was “unknown”. If both replicates had the same initial genotype call, then the combined allele call was assigned as the consensus. If one of the replicates had an “unknown” initial allele call then the genotype produced by the other replicate was assigned as the consensus. All data processing/mapping was done with a combination of Visual Basic scripts (available on request) and spreadsheet/database software.

### Description of Mapping Approach

As noted above, the genetic mapping of very large numbers of markers presents computational challenges that can far outstrip the ability of even very powerful computers to solve through exhaustive searches. The problem can, to some extent, be simplified by combining into a single locus all genetic markers that exhibit identical segregation patterns within the mapping population. As the number of markers approaches and then exceeds the number of crossovers in the “true” genetic map, the likelihood that each additional marker will match a previously observed segregation pattern increases to near certainty. Eventually, all possible genotypic patterns will be observed with each being separated from its genetic map “neighbors” by a single crossover. Each of these patterns can be defined as a “bin”, reducing the effective number of loci involved in the computation of map order. The estimation of map order thus becomes a less complex question of walking down a chain of bins with each step represented by a genetic distance of a single crossover event.

In the absence of double crossovers, this can be done by picking any bin at random to serve as the starting point (i.e., the “seed”), and then placing the two bins that differ by a single recombination before and after the “seed” bin. This is followed by extending the chain in both directions by placing unused bins adjacent to the ends of the growing chain until no unused adjacent bins exist, at which point the ends of the chromosome will have been reached. A new seed from the remaining, unused bins is then picked to initiate the construction of the next chromosome, and the process is repeated until all markers/bins have been ordered into chromosomal linkage groups. Rarely, an individual bin can appear to be adjacent to three other bins due to the occurrence of an initial crossover event followed by an adjacent crossover in the same line. In such cases, the bin in question is re-used and a small amount of ambiguity is introduced, albeit at a highly localized scale. When all possible bins are represented, there is no need to calculate LOD scores to estimate the likelihood that the resulting map order is correct. Even in the rare cases in which bins are re-used, the localized orders differences are equally likely, rendering LOD calculations moot. This further reduces the computational demands.

With actual data, even with ultra-dense genotypic information, all possible bins may not be observed due to the occurrence of recombinational hotspots or genomic regions that are identical-by-descent between the parents of the mapping population (and thus lacking in mappable polymorphisms). However, when the number of loci on the map exceeds the number of bins by several-fold, the majority of bins are represented. In the case where a gap of two recombination events in separate lines/genotypes occurs between observed bins, the missing bin can only be one of two possible patterns and the number of possible missing bins for other short intervals is similarly low.

A template map based on the Illumina SNP map developed from this same population (as part of the development of the consensus map; Bowers et al. 2012) was used to produce a preliminary map template containing genetic bins into which the Affymetrix data could be placed. This genetic map template was initially based on 35 individuals for which we had both Illumina and Affymetrix data. It was then extended and improved based on the Affymetrix data to subdivide intervals where multiple recombinations occurred in the template map through the addition of 34 lines that were not included in the production of the Illumina map but were tested on the Affymetrix chips. The template map was compared to the Affymetrix results to refine it and improve it through several iterations to obtain a final template.

The resulting template map can be used to identify features on the Affymetrix chip that correspond to genetic locations because a match (or even a near match) to the template map is exceedingly unlikely to occur by chance alone. The final version of the template map consisted of 2,532 haplotype patterns out of a possible 2^69^ (≈5.9×10^20^) combinations that could be obtained from 69 ordered lines. Some recombination patterns that theoretically exist in the mapping population were not observed when two or more recombination events occurred between observed haplotypes. These gaps were no larger than 2–5 recombination events between adjacent haplotypes in the template. Because the total number of recombination events in the template can be computed (1,936), the number of unobserved patterns can likewise be computed (789). The failure to observe a pattern in the template should not prevent the mapping of any previously missing haplotypes that fall into the resulting gap. This is because, even in the largest observed gap (which spans five recombination events) the previously missing haplotype could contain no more than two differences from one of the haplotypes flanking the gap.

The genotype scores for all 2,389,589 features on the Affymetrix chip (2,372,825 sunflower sequences and 16,773 random controls) were compared to the genetic map template, and the most similar template map position as well as the number of differences from the most similar template position were determined for each feature. The minimum number of mismatches to the template and the number of lines scored (i.e., the amount of non-missing data) were then compared to the threshold determined below to decide whether or not a particular feature that could be placed on the genetic map.

### Identification of Loci That Could be Reliably Mapped

When testing millions of features simultaneously, as we have done above, the informatics task becomes a question of separating the subset of features that can be reliably mapped from the majority of other features that are unmappable due to a lack of differentiation between the parents, hybridization to multiple copies resulting in overly complex segregation patterns, etc. We thus sought to identify the characteristics of mappable features and determine how they can be distinguished from those that primarily produce statistical noise. To do this, we identified 6,984 SNPs from the previously developed Illumina SNP (consensus) map that could be mapped uniquely to a single position on one or more of the component maps. A subset of 5,302 of these loci were targeted by 8,326 probe sets (265,376 features total) in the Affymetrix chip design, thereby providing us with a set of (mostly) single-copy loci that were tested with both technologies and whose presumptive map positions were previously known. In some instances, these presumptive single-copy genes were targeted by multiple probe sets because the initial assembly included data from multiple species and divergent alleles were sometimes assembled into separate unigenes. The best-fit map locations for all 265,376 of these features were determined from the Affymetrix data and compared to the Illumina results for these same genes. Instances in which features mapped within 10 cM of the targeted Illumina SNP were considered a match, while others were considered non-matching. The extent of agreement between the Affymetrix and Illumina data was computed as a function of the number of lines scored (i.e., the amount of non-missing data) and the minimum number of mismatches to the genetic map template.

### Simulated Data

A simulation consisting of 1,400,000 randomly generated segregation patterns using the average observed genotype frequencies was compared to the map template to determine a false discovery rate (i.e., the likelihood that a randomly generated segregation pattern would match the template by chance) based on number of lines scored and the number of mismatches to the template. The threshold for “high quality” (i.e., reliably mappable) data was set such that only 1% of the features below this threshold would be expected to match the template by chance alone.

## Results and Discussion

The Affymetrix GeneChip designed herein contains millions of features corresponding to sunflower gene sequences. Because these features were not specifically designed to target known polymorphisms, the vast majority were not expected to reliably differentiate the parents of our mapping population. For the subset that were different between the two parents of the mapping populations due to genetic polymorphisms and which corresponded to a single genetic location, only a subset are expected to be able to be scored reproducibly. The reproducibility of the scoring could be affected by background hybridization, reduced but not eliminated hybridization signal caused by the genetic polymorphism, and/or the experimental variation resulting from assaying microscopic quantities of DNA.

All 2,389,915 features on the chip were assigned a best-hit map position based on similarity to segregation patterns used in the construction of the template map. Features that were monomorphic or reflected complex multi-copy segregation patterns would be expected to have fewer individuals that could be assigned genotypes due to disagreement between the replicated chips and should exhibit a low level of concordance with the template map (i.e., no better than expected by chance). Conversely, features corresponding to distinct sequences that segregated as individual loci would be expected to have a much higher fraction of individuals that could be assigned genotypes, and should match the template map if scored correctly. Between these extremes are features that may correspond to segregating polymorphisms but which produce hybridization signals that are difficult to interpret. For example, the polymorphic hybridization signal could be superimposed on a higher level of background hybridization or the polymorphism could produce only a slight change in hybridization intensity.

The distribution of the number of plants scored for each feature and the minimum number of differences versus the template is shown in (Dataset S1). Only a small fraction of features (190 of 2,389,915 total) could be scored for all 69 plants with an exact match to the template. Beyond these 190 features, the number of plants that could be scored decreased (i.e., missing data increased) and the number of differences versus the template increased. In some cases, this was due to a lack of polymorphism accompanied by stochastic variation. In other cases, the signal resulting from the underlying genetic polymorphism, the strength of which varied across features, was increasingly blurred by background noise resulting in a gradient of detectability.

Approximately 11% (265,376) of the features on the Affymetrix chip corresponded to sequences that had previously been mapped as single copy genes in the sunflower consensus map [5], which was constructed using an Illumina Infinium array targeting 9,480 individual SNPs [6]. The distribution of these features with regard to number of plants scored and number of mismatches relative to the template (Dataset S2) was similar to the overall distribution of all features (Dataset S1). When the inferred map position of each of these features was compared between the two technologies it was found that less than 10% (25,938) mapped to the similar locations (i.e., within 10 cM of each other on the same chromosome in the corresponding maps). This subset of features was clearly biased towards higher numbers of plants scored (i.e., less missing data) and fewer mismatches versus the template (Dataset S3). Indeed, for cells in the lower left of the overall distribution, anywhere from 70% to as high as 94% of the features mapped to equivalent locations in the two maps. For features with fewer plants scored and larger numbers of mismatches, only 2–3% of the features mapped to the same location between the two maps. Taken as a whole, the map positions of this latter set of features appear to be essentially random relative to the true map position based on the high quality Illumina data.

The frequency with which the Affymetrix and Illumina results correspond increases along a gradient from the top right to the bottom left of Dataset S4. Between the extremes there exists a region where the Affymetrix features become increasingly difficult to score and genetic map placement becomes less reliable. Based on our simulations of 1,400,000 random loci (Datasets S4, S5), a threshold that contained only 0.03% of the random data was set as the cutoff for “high quality” data. This threshold is indicated by a solid line in Datasets S2, S3, S4. There are 67,846 features beyond this threshold in the full Affymetrix data set, while only ca. 700 features would be expected to fall beyond the threshold by chance. We thus estimate that ca. 1% of the 67,846 features that lie beyond the threshold are likely to be false positives (i.e., assigned to a map position due to chance similarity between their observed segregation pattern and the template map).

**Figure 1:**
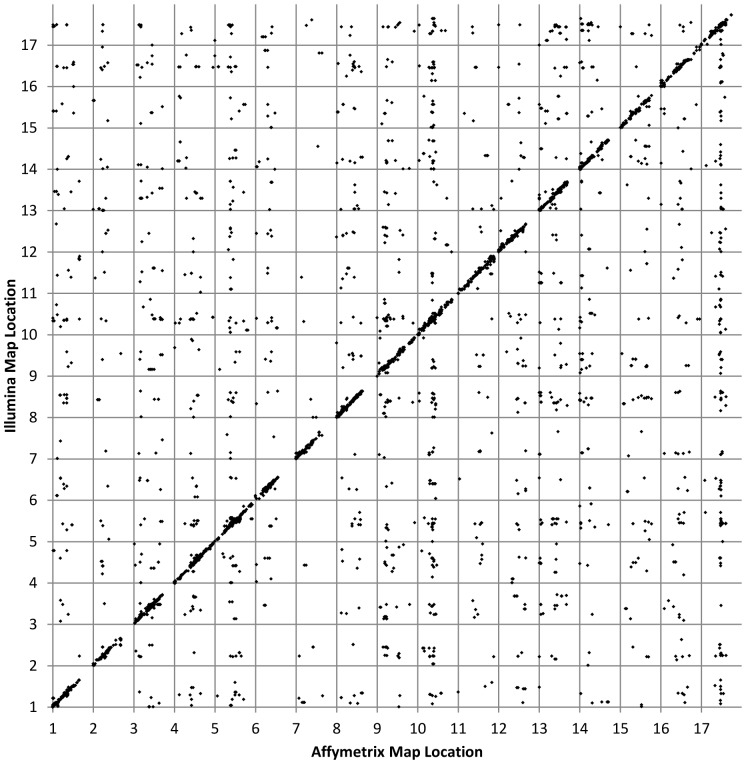
Comparison to published SNP map. Includes 11,750 loci on the Affymetrix map that matched by Blastn to sequences that mapped to a single location on the sunflower consensus map [5]. 9,239 (78.6%) of these features mapped to syntenic locations between the two maps.

When this empirical threshold was applied to the features that could be compared between the Affymetrix and Illumina maps, 11,750 of the 265,376 features fell beyond the threshold and are thus considered to represent good data. Inspection of Figure 1 reveals that nearly 80% of these features mapped to syntenic positions (i.e., on the same chromosome and within 10 cM of each other) on the corresponding maps. While some of these loci do not agree in position between the two datasets, some level of disagreement is expected due to the highly duplicated nature of the sunflower genome [16], and some duplicated genes may appear single copy with the highly targeted Illumina detection system. In fact, 12% of the SNPs on the earlier Illumina map were found to map to different locations in different crosses despite hybridizing to an identical probe targeting a single SNP. This result is consistent with the existence of a substantial number of relatively close paralogs within the sunflower genome. The ca. 20% of loci that disagree between the Illumina and Affymetrix results is thus not surprising because the probes on the Affymetrix map were matched to the original Illumina probe sequences via BLAST as the best hits from the (different) 50,020 unigene assembly used to construct the Illumina chip. As such, the true match to the sequences on the Affymetrix chip may not have been present in the EST assembly used on the Illumina chip. Rather, an unknown number of these probes are likely to have been derived from paralogous sequences. Interestingly, for features designed from *H. annuus* sequences, 83% mapped to congruent positions in the two studies. In contrast, of the features derived from sequences of other *Helianthus* species, a lower fraction (64%) mapped to congruent positions. This may be reflective of duplication events in *H. annuus* that post-date the split from other *Helianthus* species, in which case the “other species” sequences would be equally diverged from the *H. annuus* copies.

**Figure 2:**
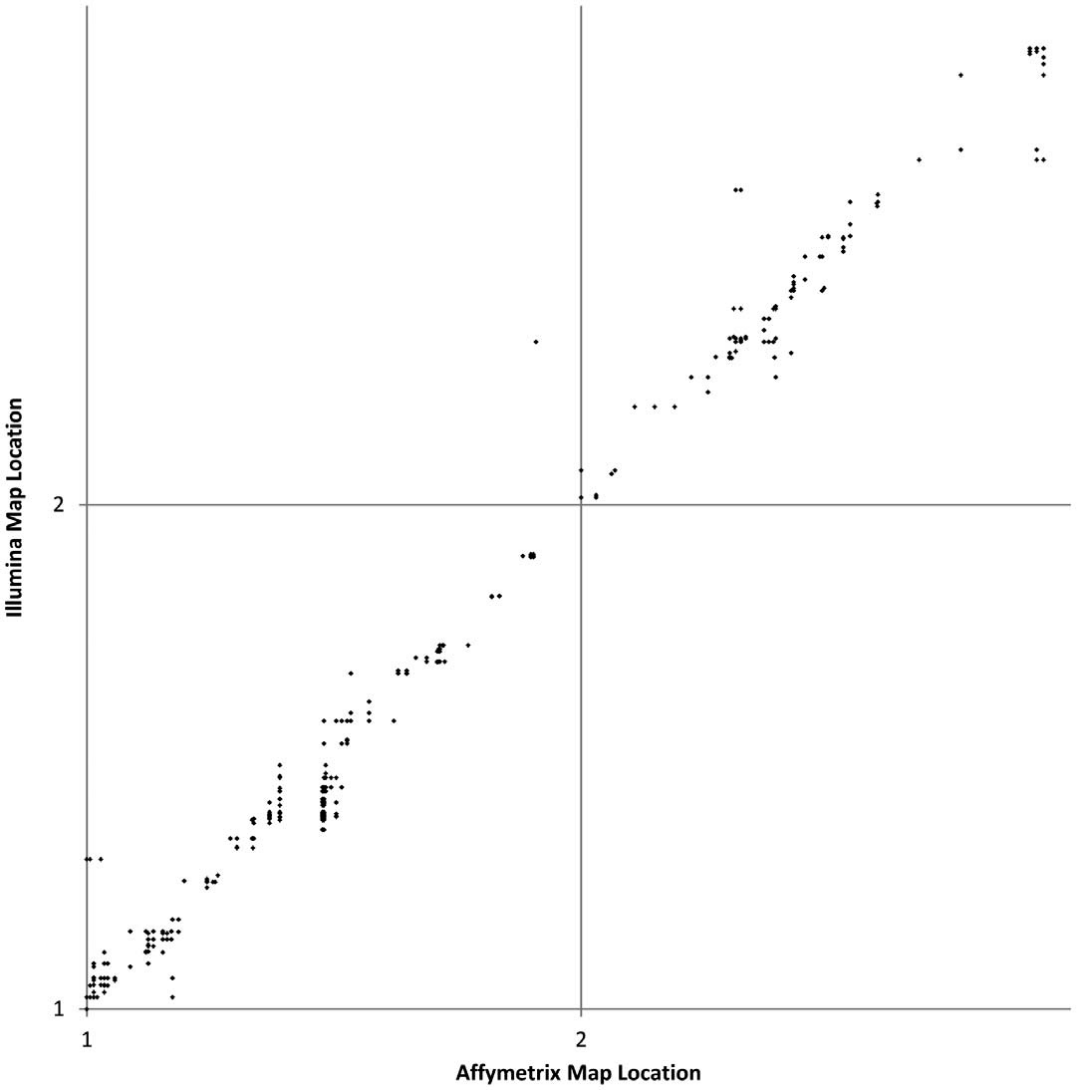
Comparison to published SNP map for linkage groups 1 and 2. Detailed comparison of map positions along linkage groups 1 and 2 based on 944 loci on the Affymetrix map that matched by Blastn to sequences that mapped to a single position on the sunflower consensus map [5].

At a more localized scale, the agreement between the Affymetrix and Illumina data for this subset is not as precise as the global comparison might indicate (Figure 2). As most of the features on the Illumina map had missing data for several lines, individual features within the same set often mapped 0–5 recombination units apart when the crucial plants needed to precisely assign a locus to that genetic bin were either missing or scored incorrectly. However, the data were sufficient to place the loci in approximately the correct genetic location. It is also worth noting that the Illumina consensus map was based on the integration of four separate maps; as such, there is an assumed level of local uncertainty when it comes to ordering on this map, as well.

Out of 2,389,915 features on the Affymetrix chip 67,846 (2.39%) features could be mapped using the established criteria. The success rate of probes designed from *Helianthus annuus* sequences (singletons in the assembly) was slightly higher (8,806 out of 241,019 tested or 3.65%) than the probes designed from multi-sequence (nearly all multi-species) contigs (34,256 out of 1,040,987 tested or 3.29%). Though these numbers appear similar, the difference is highly significant (*P*<0.001) due to the extremely large sample sizes. The probes designed from sequences derived from singleton reads of other *Helianthus* species (24,774 out of 1,067,409 or 2.32%) had the lowest success rate. Of the random sequence controls, only 10 of 17,254 (0.06%) features mapped. Interestingly, the GC content of the sequence features on the chip was predictive of the success rate (*P*<0.001; Table 1). The highest success rate (3.86%) occurred when 11 of the 25 nucleotides in the sequence were G or C and success rates declined to less than half of the maximum when less than 9 or more than 14 of the nucleotides in the probe were G or C. Fortunately, the range of 9 to 14 C or G nucleotides represented the majority (81%) of the features on the chip.

**Table 1:** Distribution of mapped features by G/C content.

# of G or C bases out of 25	All features	% of features	Mapped features	% mapped
3	153	0.01%		0.00%
4	2984	0.12%		0.00%
5	12921	0.54%	3	0.02%
6	36344	1.52%	46	0.13%
7	82069	3.43%	479	0.58%
8	157852	6.60%	2450	1.55%
9	261201	10.93%	7213	2.76%
10	361241	15.12%	12908	3.57%
11	415719	17.39%	16058	3.86%
12	394558	16.51%	14311	3.63%
13	308368	12.90%	8878	2.88%
14	193965	8.12%	4082	2.10%
15	95584	4.00%	1138	1.19%
16	39363	1.65%	226	0.57%
17	15568	0.65%	45	0.29%
18	6187	0.26%	9	0.15%
19	2357	0.10%		0.00%
20	1318	0.06%		0.00%
21	850	0.04%		0.00%
22	622	0.03%		0.00%
23	412	0.02%		0.00%
24	270	0.01%		0.00%
25	9	0.00%		0.00%
Total	2389915	100.00%	67846	2.84%

The most frequent G/C content was 11 out of 25 bases; this was also the most likely category to produce mappable polymorphisms. With the exceptions of the extremes of 3 and 25 G/C basepairs, where sufficient numbers were not tested, the success rate in finding mappable features for GC content other than 11/25 was significantly lower than for 11/25 basepairs.

**Figure 3:**
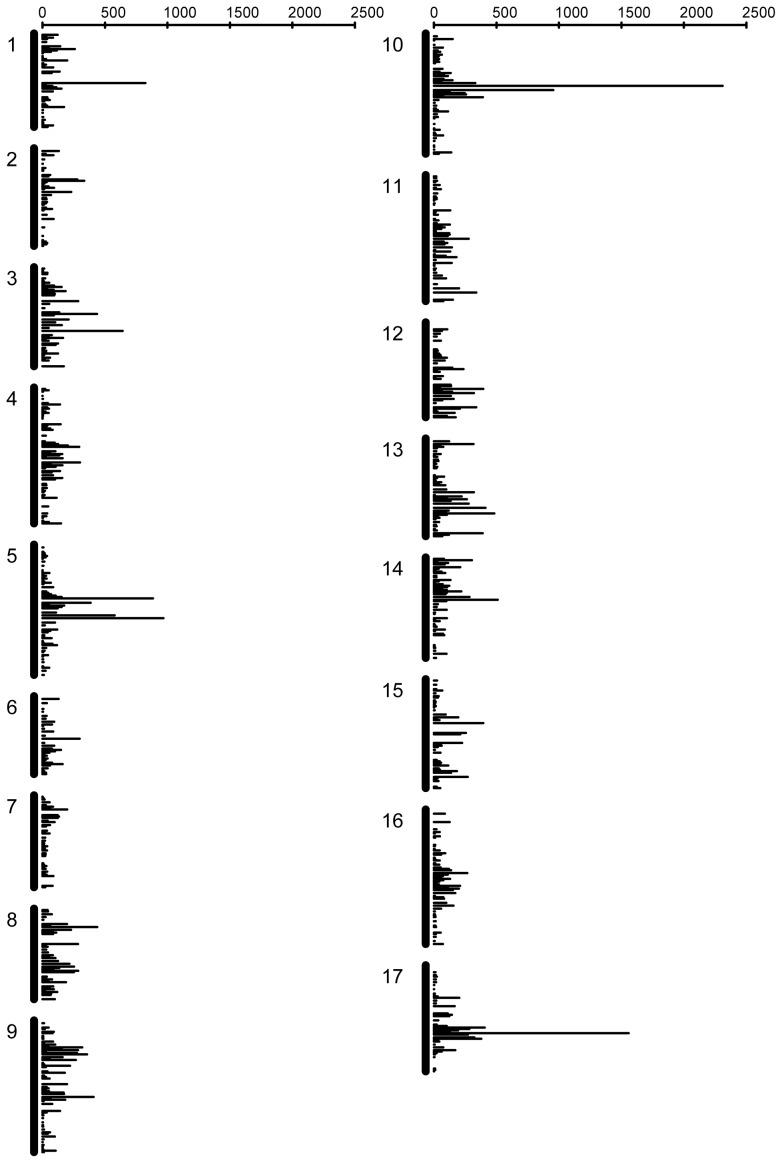
Marker density on genetic map. Marker density per cM across all 17 sunflower linkage groups. Bars represent the number of chip features mapped as SFPs using the Affymetrix technology.

The 67,846 features that were successfully mapped came from 22,481 unigenes in the original assembly, with an average of just over 3 mappable features per unigene (see Dataset S6 for a compilation of the mapping results). The map covered all 17 linkage groups without any large gaps, but marker density per cM was highly variable (Figure 3). A similar pattern was observed by Bowers et al. (2012) in this same mapping population. The regions of high marker density presumably reflect regions with high gene density and/or low recombination rates. For 19,733 of the 22,481 unigenes (ca. 88%) there were multiple features mapped per unigene. In the majority of these cases (13,660 of 19,733; ca. 70%), all features mapped congruently (i.e., within 10 cM of each other on the same linkage group). In the extreme, as many as 48 different features from the same unigene mapped congruently. Of these 13,660 loci, 765 unigenes were mapped on the basis of 10 or more features. In ca. 12% of cases (2,748 of 19,733 unigenes) different features from the same unigene mapped to more than one location. Of the unigenes that mapped to multiple locations, the majority (2,479) mapped to two locations, with 244 mapping to 3 locations, 23 mapping to 4 locations, and 2 mapping to 5 locations. Instances in which unigenes mapped to more than one location could reflect genes that have either moved with respect to their genomic position in RHA280 versus RHA801 or, perhaps more likely, they represent genes that exist in multiple copies in one or both parental lines. In the latter case, the presumption would be that a subset of features was polymorphic in one paralog with a different subset being polymorphic in one or more other paralogs. It is worth noting that the frequency of unigenes with multiple map positions in this study (ca. 12%) was similar to the frequency of SNP loci with multiple map positions when comparing amongst different crosses in our prior study using the Illumina platform (ca. 11%). Combined, these two observations can be used to provide an estimate the frequency of recently duplicated non-tandem gene sequences in sunflower of around 11–12%.

Counting multiple features that were designed based on the same unigene, and which mapped to the same location, the final map contained a total of 25,526 loci from 22,481 unigenes. Due to the multi-species nature of the sequence assembly used to design the Affymetrix chip, some of the unigenes presumably came from homologues of the same gene from different species (i.e., they represent divergent alleles of the same gene) and the true number of unique gene loci mapped is lower than this total, although how much lower is difficult to ascertain based on existing information. Remarkably, for the 67,846 features that were successfully mapped, there was missing data for an average of 17 out of 69 lines (24.7%) and an average of 3.4 lines (4.9%) produced data that did not agree with the template map and represent likely errors in the raw data. This high level of missing and erroneous data all but precluded map construction using more traditional methods. The new approach described herein was especially necessary considering that the mappable polymorphisms were mixed with over 2 million additional features that were either monomorphic or produced variation in hybridization intensity that could not be reliably distinguished from background noise.

We have demonstrated the ability to successfully map a large number of genetic loci even in the presence of relatively high error rates in the raw data. We did this by establishing a template representing most of the bins present on the true map using a lower throughput but less error prone approach and then matching observed segregation patterns to their best fitting location on the template. By matching against pre-defined bins, we were able to avoid the incorporation of errors such as additional recombination events on the map, which would dramatically inflate map distances and make them more reflective of marker numbers than the true size of the underlying genetic map. Our approach of rigorously filtering the data thus allowed us to maintain map accuracy while taking advantage of a much higher throughput (and lower cost per data point) genotyping approach and minimizing computational intensity. Importantly, this approach is robust to the relatively high error rates and potentially high frequency of non-polymorphic assays that may accompany such technologies.

Going forward, the resulting map has the potential to positively impact multiple lines of sunflower research. While it could be argued that the slight uncertainty regarding precise map locations within this map will limit its utility for genome assembly, this is not necessarily the case. Indeed, assuming the availability of a good scaffold-level assembly (with scaffolds spanning several megabases), a relatively modest number of scaffolds needs to be assembled into chromosomes to produce a genome-level assembly. Because approximate map positions can be combined with synteny information from other species, a physical map, and long insert paired-end sequences to assemble a high quality genome [17], this map represents a valuable resource for the eventual assembly of the sunflower genome. Because it contains a substantial fraction of all genes in the sunflower genome, this map will also serve as a valuable tool for the identification of candidate genes underlying QTL or traits that have been mapped in other studies.

## Supporting Information

Dataset S1 (XLSX)

Dataset S2 (XLSX)

Dataset S3 (XLSX)

Dataset S4 (XLSX)

Dataset S5 (XLSX)

Dataset S6 (XLSX)
